# Supplementary material for: Baseline Characterization of the Gut Microbiota of Field and Colony Populations of Phlebotomus tobbi and Preliminary Assessment of the Anti-Leishmanial Activity of Cultivable Bacteria
Source: Pathogens. 2026 Jun 23;15(7):658. doi: 10.3390/pathogens15070658 (PMC13416292; doi:10.3390/pathogens15070658)
Supplement: Supplementary file 1 [file pathogens-15-00658-s001.zip › pathogens-4357168-File S1/Taxa2ASV_Decomposer/Genus/g__Erwinia/heatmap_with_clustering.pdf]

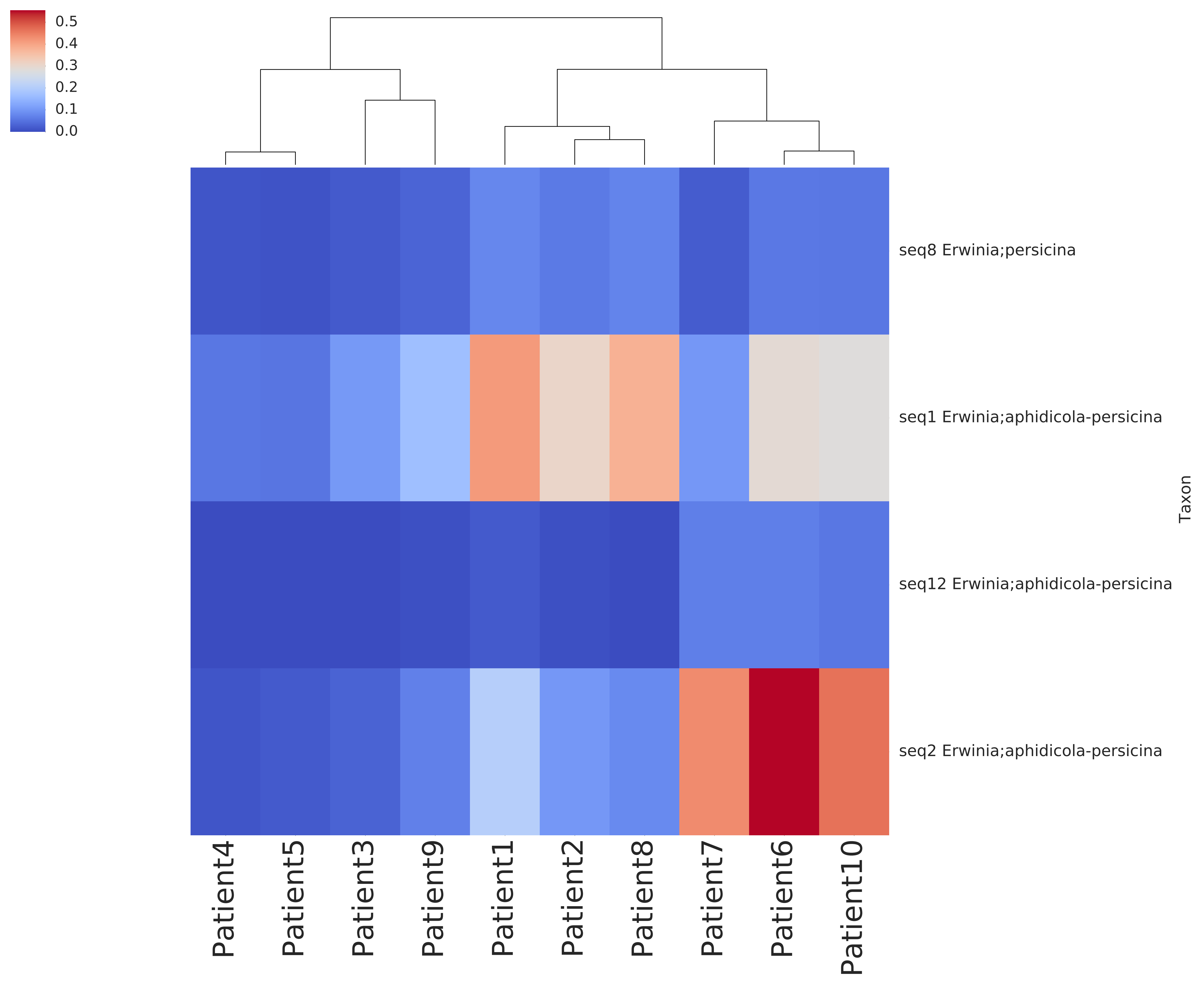

0.5
0.4
0.3
0.2
0.1
0.0
seq8 Erwinia;persicina
seq1 Erwinia;aphidicola-persicina
seq12 Erwinia;aphidicola-persicina
seq2 Erwinia;aphidicola-persicina
Taxon
Patient4
Patient5
Patient3
Patient9
Patient1
Patient2
Patient8
Patient7
Patient6
Patient10
